# Combined Analysis of the Effects of Exposure to Blue Light in Ducks Reveals a Reduction in Cholesterol Accumulation Through Changes in Methionine Metabolism and the Intestinal Microbiota

**DOI:** 10.3389/fnut.2021.737059

**Published:** 2021-11-25

**Authors:** Daiyang Xia, Lin Yang, Jiajie Cui, Yu Li, Xianzhi Jiang, Giuseppe Meca, Shunxiang Wang, Yan Feng, Yujie Zhao, Jiangfan Qin, Yongwen Zhu, Hui Ye, Wence Wang

**Affiliations:** ^1^Guangdong Provincial Key Laboratory of Animal Nutrition and Regulation, College of Animal Science, South China Agricultural University, Guangzhou, China; ^2^Microbiome Research Center, Moon (Guangzhou) Biotech Co. Ltd., Guangzhou, China; ^3^Laboratory of Food Chemistry and Toxicology, Faculty of Pharmacy, University of Valencia, Valencia, Spain; ^4^Guangdong Haida Group Co. Ltd., Guangzhou, China; ^5^Gold Coin Feedmill (Dong Guan) Co. Ltd., Dongguan, China; ^6^Cofco Feed (Foshan) Co. Ltd., Foshan, China

**Keywords:** blue light, methionine metabolism, intestinal microbiota, cholesterol, bile acid

## Abstract

Monochromatic light is widely used in industry, medical treatment, and animal husbandry. Green-blue light has been found to stimulate the proliferation of satellite cells and the results of studies on the effects of blue light on poultry vary widely. It would be worthwhile to study the effect of blue light on poultry growth and how exposure to blue light affects metabolism and the intestinal microbiota. In this study, we irradiated Cherry Valley ducks with 460 nm wavelength light (blue light) for 3 weeks to explore the effects of blue light in comparison to those of white light (combined wavelength light) on animal growth and development. Our results showed that, under exposure to blue light, the body weight and average daily feed intake of ducks were decreased, but the leg muscle and relative length of the intestine were increased. Exposure to blue light chiefly enhanced the anti-inflammatory and antioxidant capacities of the animal and decreased lipid levels in serum and liver. Metabolomic analysis revealed that blue light heightened cysteine and methionine metabolism, and increased serum taurine and primary bile acid levels, as well as up-regulating the metabolites L-carnitine and glutamine. Treatment with blue light significantly increased the beta diversity of intestinal microbiota and the relative abundances of bile acid hydrolase-producing bacteria, especially *Alistipes*. These changes promote the synthesis of secondary bile acids to further enhance lipid metabolism in the host, thereby reducing cholesterol accumulation in ducks. These results should help us better understand the effects of exposure to blue light on metabolite levels and the intestinal microbiota, and suggest that it may be possible to use colored light to control the development of livestock and poultry.

## Graphical Abstract

Blue light improves lipid metabolism by altering metabolism and gut microbes. 
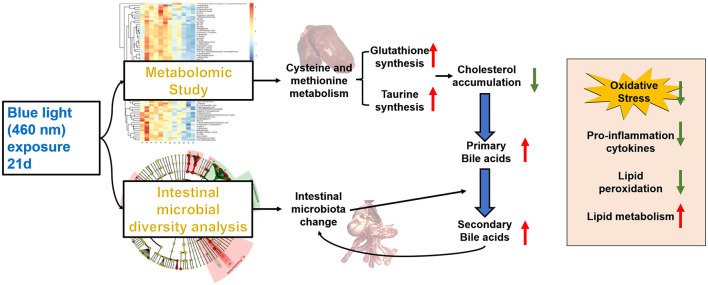

## Introduction

Illumination plays an important role in the regulation of circadian rhythm and metabolic homeostasis. Monochromatic light is widely used in animal husbandry to influence embryo development and poultry production (1). Compared with mammals, birds have a unique visual adjustment mechanism, superior visual function, and a photosensitive range that has been ascribed to ciliary muscles and four types of retinal cone cells (2–4). However, it is unknown whether avian species have an intrinsic metabolic mechanism and microbial structure under stimulation with monochromatic light. Blue light is colored light with a wavelength ranging between 400 and 500 nm (5). Due to its fruitful broad-spectrum antibacterial effect (6–8), LED blue-light irradiation has been used for the treatment of skin diseases and wound sterilization (9–11). Various studies have revealed that blue light also plays a role in regulating physiological functions *in vivo*, e.g., it improves cognitive performance (12), promotes cell proliferation and differentiation (13, 14), and even inhibits tumor cell growth and metastasis (15, 16).

Investigations of the effects of blue light on poultry have not provided definitive conclusions. Several studies have indicated that blue light and blue-green light promote growth performance and improve the fatty acid composition in Cherry Valley ducks, and green-blue light stimulated the proliferation of chicken satellite cells (17, 18). In some cases, blue light clearly reduced the body weight of ducks at ages 1–35 days (19). However, whether and how exposure to blue light impacts the metabolic processes of poultry is unclear.

In this study, we hypothesized that blue light may reduce growth performance and lipid deposition in ducks, and impact the composition of the intestinal microbiota. Our results revealed that treatment with blue light improved bile acid synthesis in the intestine and suppressed cholesterol accumulation. Collectively, the results of this study suggest that exposure to blue light could intensify lipid metabolism and may be exploited for the regulation of growth and development.

## Materials and Methods

### Animal Diet and Treatments

**Figure 1:**
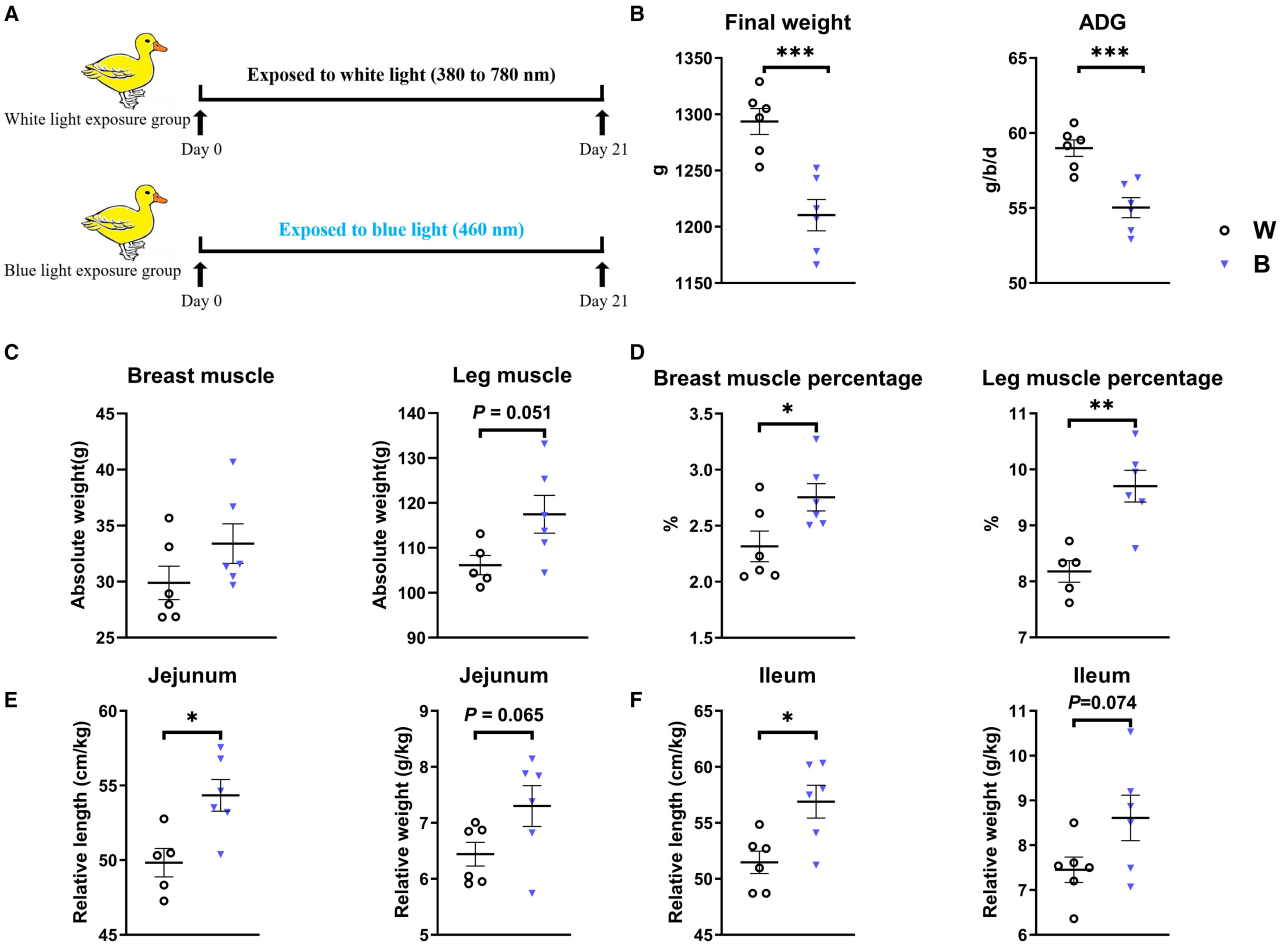
Exposure to blue light reduces body weight but increases the muscle percentage in ducklings. **(A)** Scheme of the experiments. **(B)** Effect of exposure to blue light on growth performance of ducks from days 1–21 (*n* = 6, mean with SEM). **(C)** Effect of exposure to blue light on the absolute weight and percentage of breast muscle. **(D)** Effect of exposure to blue light on the absolute weight and the percentage of leg muscle. **(E)** The effect of blue light irradiation on the relative length and weight of duck jejunum. **(F)** The effect of blue light irradiation on the relative length and weight of duck ileum. Data in **(B–F)** were analyzed using the unpaired *t*-test, *P < 0.05, **P < 0.01, ***P < 0.001.

Cherry Valley ducks (1-day-old, all male) were purchased from Foshan Guiliu Poultry Co. Ltd. (Foshan, China). A total of 120 ducklings were randomly divided into two groups, with 6 replicates for each treatment and 10 birds for each replicate; the breeding density was set to 6 per/m^2^. Birds were placed in environmentally controlled light-proof rooms separated from each other and exposed to white light (380–780 nm) and blue light (460 nm), respectively. Twenty-four hours of lighting were provided from light-emitting diode (LED) bulbs at 10 Lux for 21 days (Figure 1A). The photosynthetic photon flux density (PPFD) of the white light was 0.2 μmoL/s/m^2^ and that of blue light was 0.036 μmoL/s/m^2^ (Nuodake, Wuxi, China); LED lamp spacing 0.8 m, light-source calibration twice a week.

The body weight, feed intake, and ratio of feed to gain of the animals were regularly recorded during the treatment period. The room temperature was maintained at 32–34°C for the first 3 days, and then reduced from 2 to 3°C per week to a final temperature of 26°C. The experimental period lasted for 3 weeks. Diets were formulated to meet the China Nutrient requirements for meat-type duck (2012) (Table 1).

**Table 1:** Composition and nutrient levels of basal diets (air-dry basis, %).

**Item**	**Content (%)**
Corn	62.00
Soybean meal	31.00
Fish meal	1.00
Wheat bran	2.20
Limestone	1.20
Dicalcium phosphate	1.30
Salt	0.30
Premix^a^	1.00
Total	100
Nutrient level^b^	
ME (MJ/kg)	12.14
CP	20.00
Ca	0.87
Non phosphorous	0.42
Lysine	1.20
Methionine + cystine	0.81

a *Premixe supplied per kilogram diet: vitamin A, 12,000 IU; vitamin D, 3,000 IU; vitamin E, 30 mg; vitamin B_2_, 9 mg; vitamin B_12_, 0.03 mg; vitamin K_3_, 6 mg; pantothenic acid, 60 mg; niacin, 11 mg; folic acid, 1.5 mg; biotin, 0.18 mg; Fe, 90 mg; Cu, 8 mg; Mn, 100 mg; Zn, 70 mg; I, 0.6 mg; Se, 0.4 mg.*

b *Nutrient levels are calculated values*.

### Sample Collection

After 3 weeks of rearing, one bird of average weight from each group was selected for slaughter. Animals were sacrificed by cervical dislocation, and 10 ml of blood was collected from the jugular vein; these procedures followed the agricultural industry standard of the People's Republic of China (NY/T 3741-2020). Plasma was prepared by centrifuging the blood at 3,000 r/min for 10 min and then stored at −20°C. The middle part of liver samples was collected for molecular biological analysis after washing with phosphate-buffered saline (PBS, pH = 7.2–7.4). Samples of the duodenum, jejunum, and ileum were snap-frozen in liquid nitrogen and stored at −80°C for mRNA analysis for RT-PCR.

### Serum Biochemical Analysis

Total cholesterol (TC) and activities of catalase (CAT), total superoxide dismutase (T-SOD), total antioxidant capability (T-AOC), and glutathione peroxidase (GSH-Px) in plasma and liver contents were assayed by colorimetric methods using commercial kits (Jiancheng, Nanjing, China). Insulin-like growth factor-1 (IGF-1), adiponectin, and leptin were determined using ELISA kits according to the manufacturer's instructions (Jiancheng, Nanjing, China).

### Metabolite Extraction and LC-MS/MS Analysis

In this study, UHPLC-MRM-MS/MS was used for high-throughput metabolomics detection and analysis on duck serum samples in the white- and blue-light groups. Chromatographic conditions and mass spectrometry were performed under the conditions described at www.researchsquare.com.

### 16S rRNA Amplicon Sequencing, Data Processing, and Analysis

DNA extraction, PCR amplification, MiSeq sequencing, and processing of sequencing data were carried out as described at pubs.rsc.org.

### Pro-inflammatory Factor Measurements

ELISA kits (Nanjing Jiancheng Institute of Biotechnology, Nanjing, Nanjing) were used to measure the levels of cytokines including IL-2, IL-6, TNF-α, and IFN-γ in the liver, according to the manufacturer's instructions.

### Gene Expression Analysis

Total RNA was extracted from frozen tissue samples using a Magen HI Pure Universal RNA Mini Kit (Magen, Guangzhou, China). The first-strand cDNA was synthesized using oligonucleotide 20 and Superscript II reverse transcriptase (EZB, Guangzhou, China). Real-time PCR was performed on an ABI 7500 (Applied, Bio-Systems, Foster City, CA) using a SYBR Green Quantitative PCR Kit (TaKaRa, Japan). The mRNA expression of each gene was calculated by the 2^−ΔΔCt^ method. β-actin was used as an internal standard to normalize the transcription level of target genes. Use the following primers for transcription analysis of related genes: *ACC*: 5′-TAG CCA TGC AGC CAC TTT GA-3′, 3′-ACC TTT GTA CGA GCT GCA CA-5′, *FAS*: 5′-GGA AGT GCC CCA GTT GAA GA-3′, 3′-GCT GCA ATG CCC CAT GAT G-5′, *ChREBP*: 5′-ACA TCA TCT TGC GGC AGT GA-3′, 3′-TGT GAT ACG CCG GCT TTC TAT-5′, *FABP*: 5′-ATG AGA CCA CAG CAG ATG ACA-3′, 3′-TTT GCC ATC CCA CTT CTG CA-5′, *LPL*: 5′-AAG CTC TGC GTC TGA TTG CT-3′, 3′-TGC TGG GCT TTT CTT CGT AGA-5′, *SREBP-1*: 5′- CAT CCA TCA ACG ACA AGA TCG T-3′, 3′-CTC AGG ATC GCC GAC TTG TT-5′, β-Actin (5′- GCT ATG TCG CCC TGG ATT T-3′, 3′-GGA TGC CAC AGG ACT CCA TAC-5′) was used as an internal control to normalize the transcription level of target genes.

### Statistical Analysis

Data are shown as mean ± SEM and SAS 9.2 (SAS Institute, Inc., Cary, NC) was used for statistical analysis. Significant differences were evaluated by an unpaired Student's *t*-test or the Mann-Whitney U test on both sides of non-normally distributed samples. Differences were considered to be significant at *P* < 0.05; ^*^P < 0.05; ^**^P < 0.01; ^***^P < 0.001; and ^****^P < 0.0001.

## Results

### Treatment With Blue-Light Irradiation Promotes Muscle Development in Ducks

The body weight and feed intake of ducklings were monitored during the experiment to explore the effect of exposure to blue light (Figure 1A). We found that exposure to blue light significantly decreased the final weight (*P* = 0.001) and Average Daily Gain (ADG, *P* = 0.001) compared to those in the white-light group (Figure 1B). Interestingly, although the weight of ducklings in the blue-light group decreased, the absolute weight of their breast muscle and leg muscle tended to increase, and the ratio of breast muscle to leg muscle significantly increased (*P* < 0.05; Figures 1C,D). In addition, the relative length and weight of the jejunum of the ducklings in the blue-light group were significantly higher than those in the white-light group (*P* < 0.05), and the relative length and weight of the ileum also tended to be higher. These results demonstrate that exposure to blue light affects the growth and development of ducks (Figures 1E,F and Supplementary Figure S1).

### Treatment by Irradiation With Blue Light Improves the Anti-oxidant and Anti-inflammatory Capacities of Ducks

**Figure 2:**
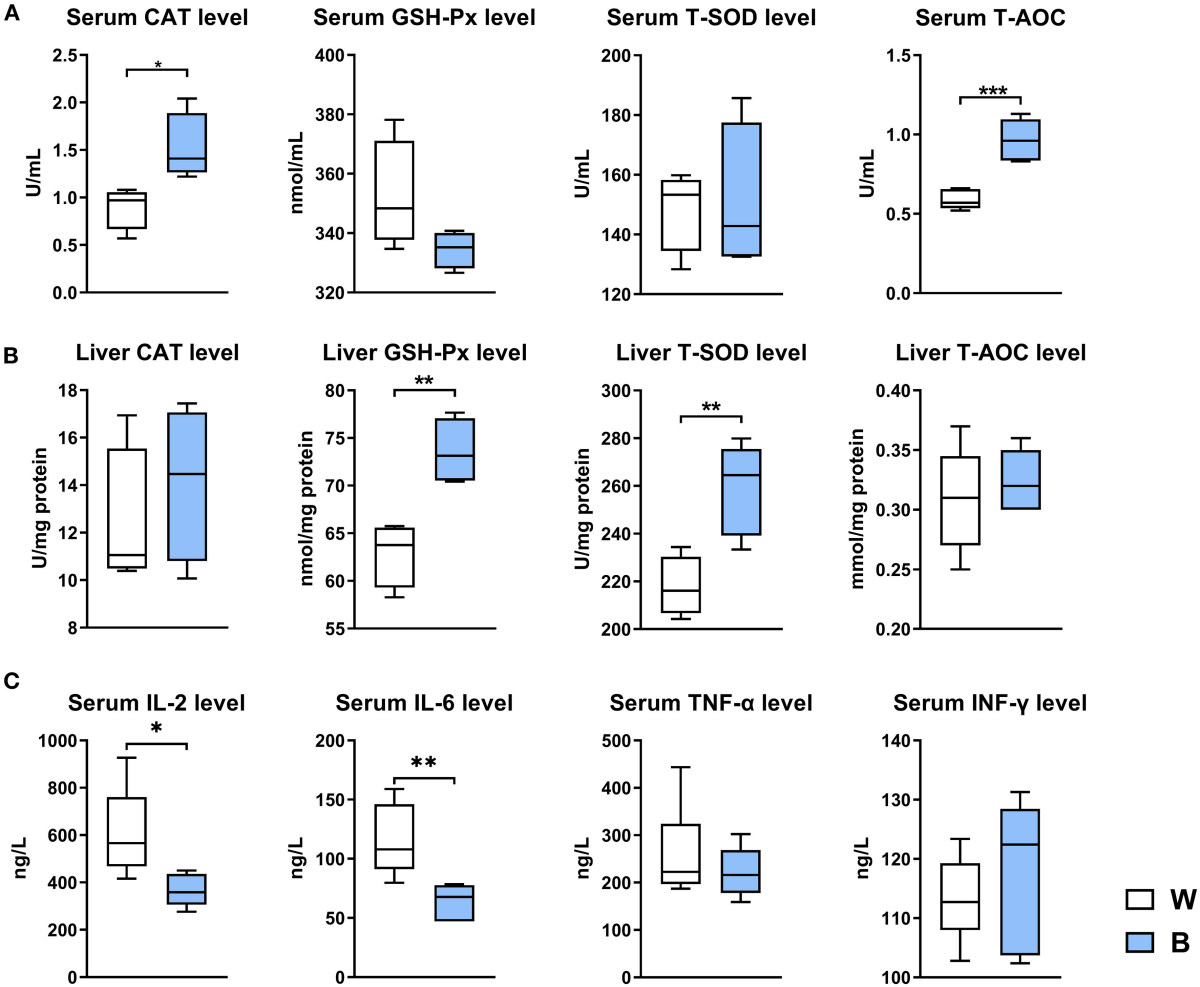
Exposure to blue light improves the antioxidant and anti-inflammatory capacities in ducks. **(A)** Effect of exposure to 460 nm LED blue light on the serum antioxidant indicators CAT, GSH-Px, T-SOD, and T-AOC in ducks (*n* = 6, mean with SEM). **(B)** Effect of exposure to blue light on the liver antioxidant indicators CAT, GSH-Px, T-SOD, and T-AOC in ducks (*n* = 6, mean with SEM). **(C)** Effect of exposure to blue light on serum cytokine levels in ducks, including IL-2 IL-6, TNF-α, and IFN-γ (*n* = 6, mean with SEM). Data in **(A–C)** were analyzed using the unpaired *t*-test, *P < 0.05, **P < 0.01, ***P < 0.001.

Exposure to blue light significantly increased serum CAT (*P* < 0.05) and T-AOC levels (*P* < 0.001), and significantly increased GSH-Px and T-SOD levels in the liver (*P* < 0.001; Figures 2A,B). We also measured the levels of inflammatory cytokines in serum, and found that exposure to blue light significantly reduced the levels of pro-inflammatory cytokines such as IL-2 (*P* < 0.05) and IL-6 (*P* < 0.001) compared to those in the white-light group (Figure 2C). In general, these results indicate that exposure to blue light could improve antioxidant and anti-inflammatory capacities in ducks.

### Treatment by Irradiation With Blue Light Improves Lipid Metabolism in Ducks

The increase in muscle weight and the decrease in body weight occurred at the same time, along with the improvements in antioxidant and anti-inflammatory capacities. These changes suggest that blue light may have an ameliorative effect on lipid metabolism. Therefore, we determined the levels of hormones in serum, the expression of genes related to lipid metabolism in the liver, and the content of cholesterol in the liver and serum.

**Figure 3:**
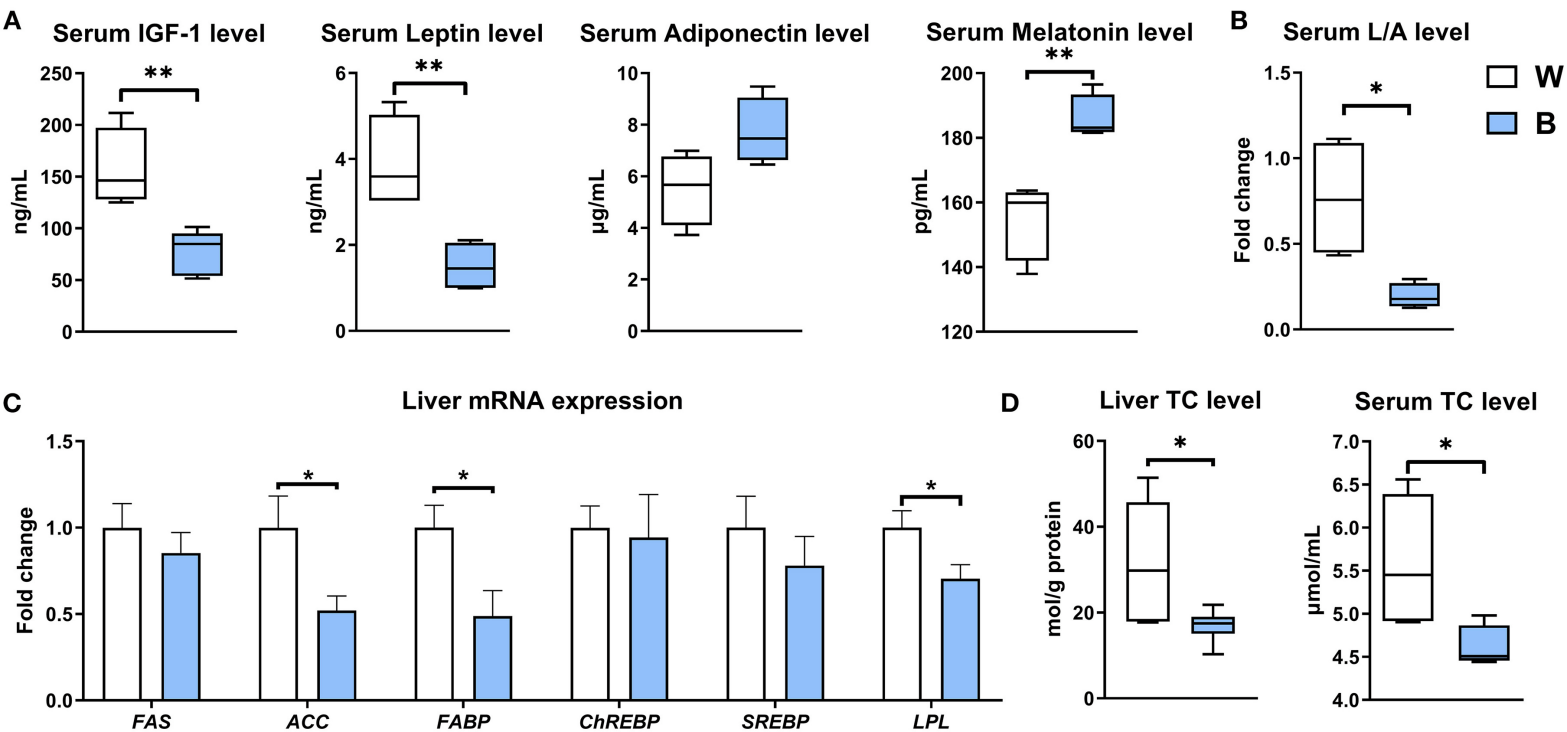
Exposure to blue light reduces the accumulation of cholesterol in the liver and serum. **(A)** Effect of blue light on serum hormone levels of IGF-1, leptin, adiponectin, and melatonin in ducks (*n* = 6, mean with SEM). **(B)** Effect of blue light on serum ratio of leptin to adiponectin in ducks (*n* = 6, mean with SEM). **(C)** Relative mRNA expression of *FAS, ACC, FABP, ChREBP, SREBP*, and *LPL* in the liver of blue light-exposed ducks (*n* = 6, mean with SEM). **(D)** Effect of blue light on the liver and serum total cholesterol levels in ducks (*n* = 6, mean with SEM). Data in **(A–D)** were analyzed using the unpaired *t*-test, *P < 0.05, **P < 0.01.

Serum IGF-1 and leptin levels were down-regulated in the blue-light group (*P* < 0.01), while adiponectin and melatonin were up-regulated (*P* < 0.01; Figure 3A); exposure to blue light reduced the ratio of leptin to adiponectin (L/A) to 25.47% of that in the white-light group (*P* < 0.05; Figure 3B). The L/A ratio is a critical index of insulin resistance and obesity, and its elevation is often accompanied by metabolic diseases (20). Blue light also significantly reduced the expression levels of genes involved in fat synthesis, including *ACC, FABP*, and *LPL*, in the liver (*P* < 0.05; Figure 3C). Furthermore, irradiation with blue light also significantly reduced the accumulation of cholesterol in duck liver and serum (*P* < 0.05; Figure 3D): the cholesterol content in the liver was reduced by 47.1%, and that in the serum was reduced by 17.8%. These results collectively suggest that exposure to blue light can improve the efficiency of energy conversion and lipid metabolism, especially cholesterol metabolism.

### Treatment by Irradiation With Blue Light Alters Cysteine and Methionine Metabolism in Ducks

**Figure 4:**
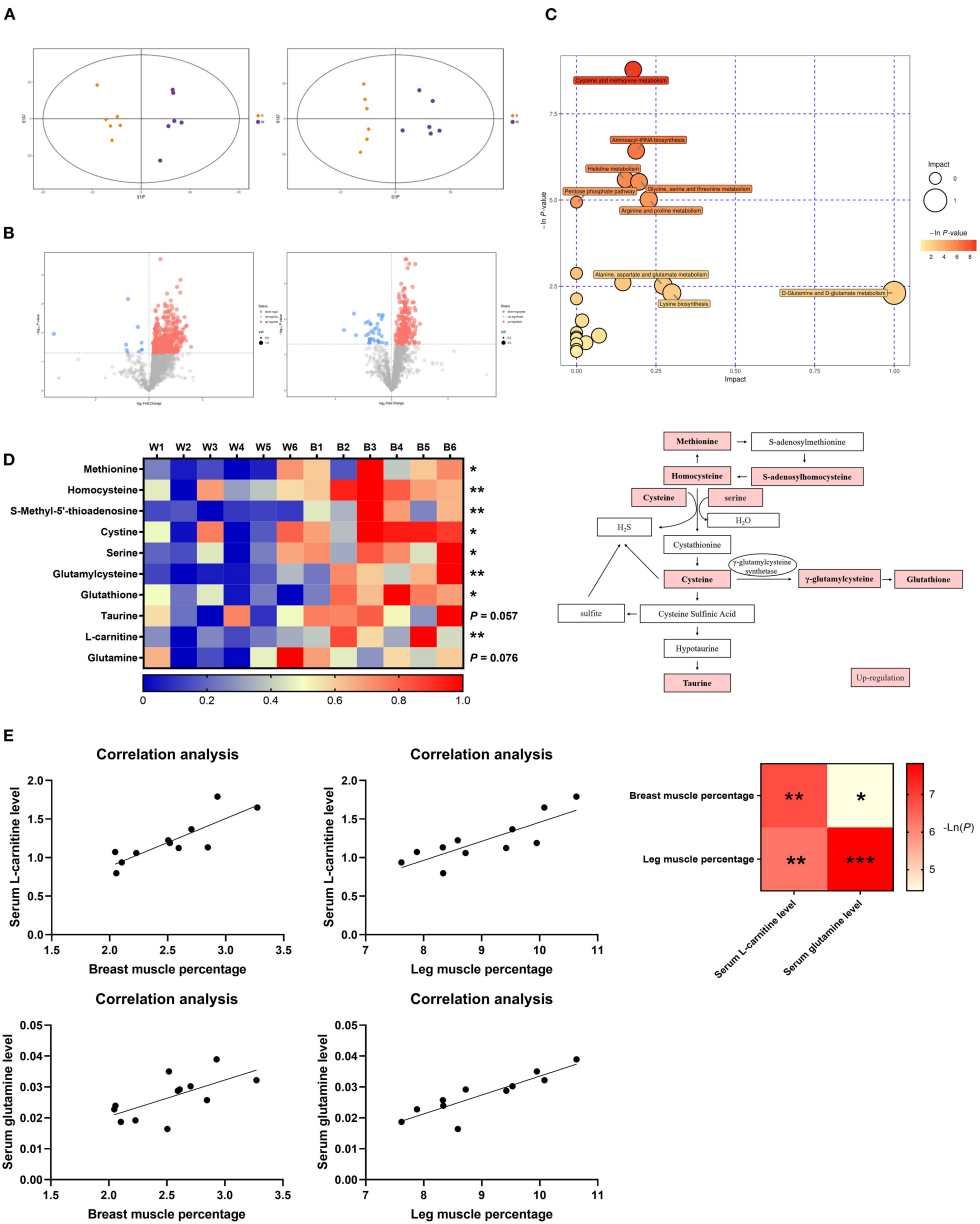
Exposure to blue light improves glutathione and taurine levels in ducks by promoting methionine and cysteine metabolism. **(A)** OPLS-DA score plots of metabolomics (*n* = 6). **(B)** Volcano plot representation of the differences in metabolites (*n* = 6). **(C)** Metabolic pathway bubble chart (*n* = 6). **(D)** Heatmap of LC-MS data showing metabolite changes after LED treatment with 460 nm illumination (*n* = 6). **(E)** Correlation analysis of the serum L-carnitine level and glutamine level with breast muscle percentage and leg muscle percentage. Increases in metabolite levels are shown in red. Data in d were analyzed using the unpaired *t*-test, *P < 0.05, **P < 0.01. Data in **(E)** were analyzed using the computer non-parametric Spearman correlation ***P < 0.001.

To further explore the underlying mechanism by which irradiation with blue light regulates lipid metabolism, we performed a non-target metabolomics analysis of serum in the two groups. In this study, we extracted a total of 1,684 and 1,933 metabolic features in the POS (Positive) mode and NEG (Negative) mode, respectively. According to the OPLS-DA analysis, there was a vast metabolic difference between the white-light and blue-light groups (Figure 4A). The results of 200 permutation tests demonstrated that the developed models do not overfit (Supplementary Figure 2A). We calculated the Euclidean distance matrix of the quantitative values of the different metabolites, calculated the aggregated differential metabolites using the full-link method, and displayed the different metabolites using a heat map (Supplementary Figure 2). A total of 105 metabolite biomarkers were significantly different between the two groups, and exposure to blue light up-regulated 102 of them, which was also consistent with volcano plots (Figure 4B). Based on the KEGG database, MetaboAnalyst (4.0) was used to analyze the most relevant metabolic pathways that were changed by exposure to blue light, and we focused on the metabolic pathways for methionine, cysteine and lysine (Figure 4C).

As an essential precursor of cellular methylation, methionine is an indispensable substance in various physiological and biochemical processes (21). According to the pathway analysis, most of the metabolic biomarkers in this pathway were up-regulated (Figure 4D), which suggested that cysteine and methionine metabolism were positively associated with exposure to blue light in ducks. Compared to the white-light group, levels of methionine, homocysteine, cysteine, serine, γ-glutamyl cysteine, and glutathione were elevated (*P* < 0.05), with fold-changes of 1.96, 1.21, 1.38, 1.60, 2.06, and 2.02, respectively, in the blue-light group. Notably, compared to that in the white-light group, the taurine content in the blue-light group was also increased (*P* = 0.057). Under exposure to blue light, the metabolic pathways of lysine were also upregulated (Figure 4C). As a downstream metabolite of methionine and lysine, the L-carnitine level was significantly up-regulated [*P* < 0.01; (22)]. The serum glutamine content also tended to increase (*P* = 0.076; Figure 4D). Since both of these metabolites are associated with fat metabolism and muscle production, we analyzed the correlation between them and the muscle percentage. L-Carnitine and glutamine levels were positively correlated with the breast muscle rate and leg muscle percentages (Figure 4E).

In summary, exposure to blue light can significantly promote the metabolism of cysteine and methionine in ducks, leading to an increase in the content of the final metabolites: glutathione and taurine.

### Treatment by Irradiation With Blue Light Promotes the Synthesis of Bile Acids

**Figure 5:**
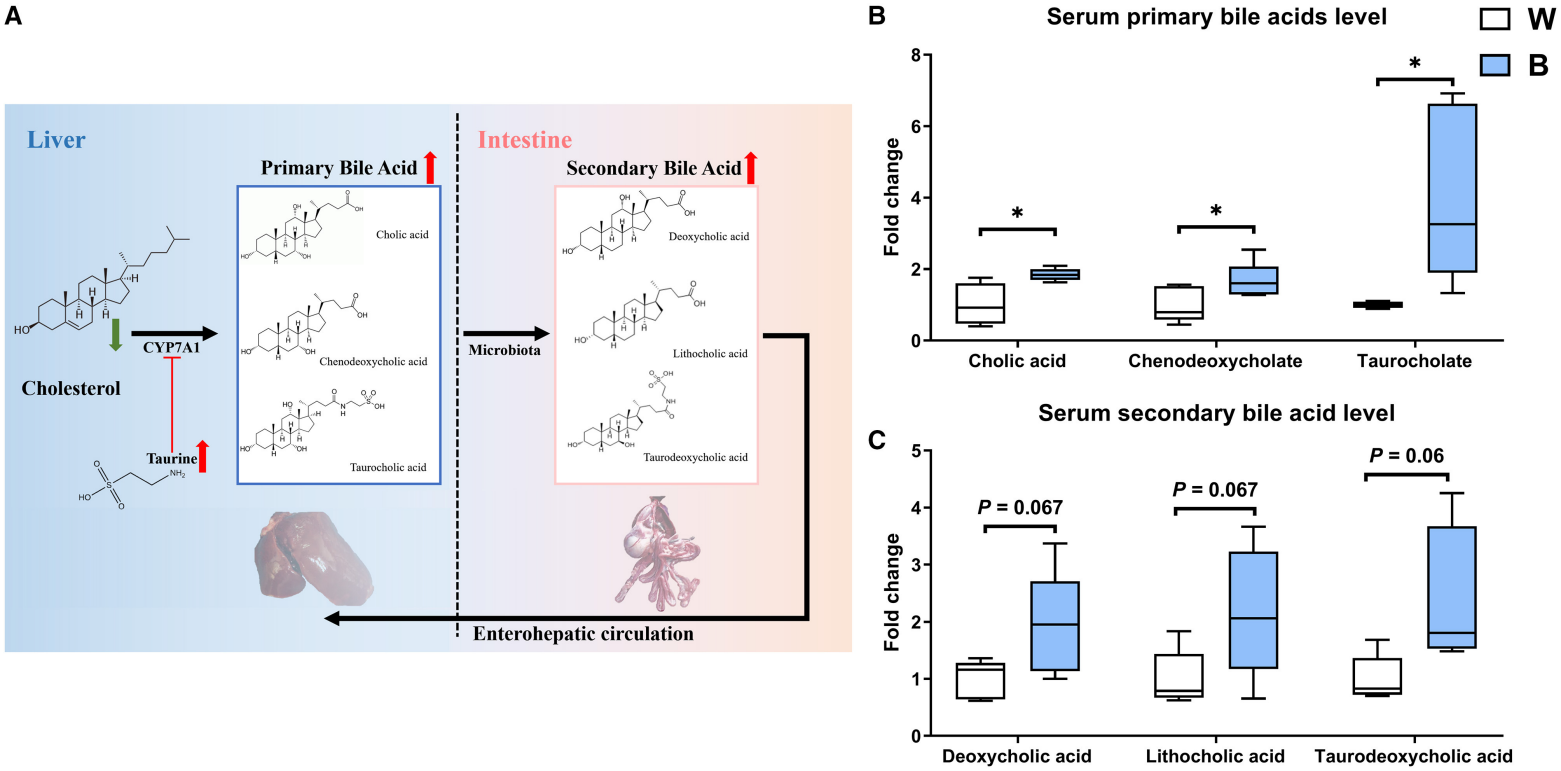
Exposure to blue light promotes bile acid synthesis. **(A)** Effects of blue light on synthesis and secretion of primary and secondary bile acids in ducks. **(B)** Effects of blue light on primary bile acid levels of cholic acid, chenodeoxycholic acid, and taurocholic acid in ducks (*n* = 6, mean with SEM). **(C)** Effects of blue light on secondary bile acid levels of deoxycholic acid, lithocholic acid, and taurodeoxycholic acid in ducks (*n* = 6, mean with SEM). Data in **(B,C)** were analyzed using the unpaired *t*-test, *P < 0.05.

Glutathione and taurine are strongly correlated with antioxidant function (23, 24). This would explain the enhanced anti-oxidation capacity in the blue-light group mentioned above. Here, we examine the relationship between taurine and cholesterol metabolism (25). Taurine can improve the activity of cholesterol 7α-hydroxylase (CYP7A1), which converts cholesterol into primary bile acids (26) (Figure 5A).

Exposure to blue light significantly increased primary bile acid levels (e.g., cholic acid, chenodeoxycholate, and taurocholate). In particular, the taurocholate level in the blue-light group was almost four times higher than that in the white-light group (*P* < 0.05; Figure 5B). Notably, compared to the white-light group, secondary bile acid levels (e.g., deoxycholic acid, lithocholic acid, and taurodeoxycholic acid) were elevated in the blue-light group, with fold-changes of 1.928, 2.173, and 2.335, respectively (Figure 5C).

### Exposure to Blue Light Alters the Intestinal Microbiota in Ducks

Secondary bile acids are synthesized and metabolized in the intestine and are dominated by the intestinal microbiota. As mentioned above, the content of secondary bile acids in the blue-light group tended to be up-regulated, so the influence of blue light on intestinal microbes should also be considered. To illustrate the interaction between the intestinal microbiota and bile acids, we analyzed the cecum microbiota by 16S rRNA gene sequencing.

**Figure 6:**
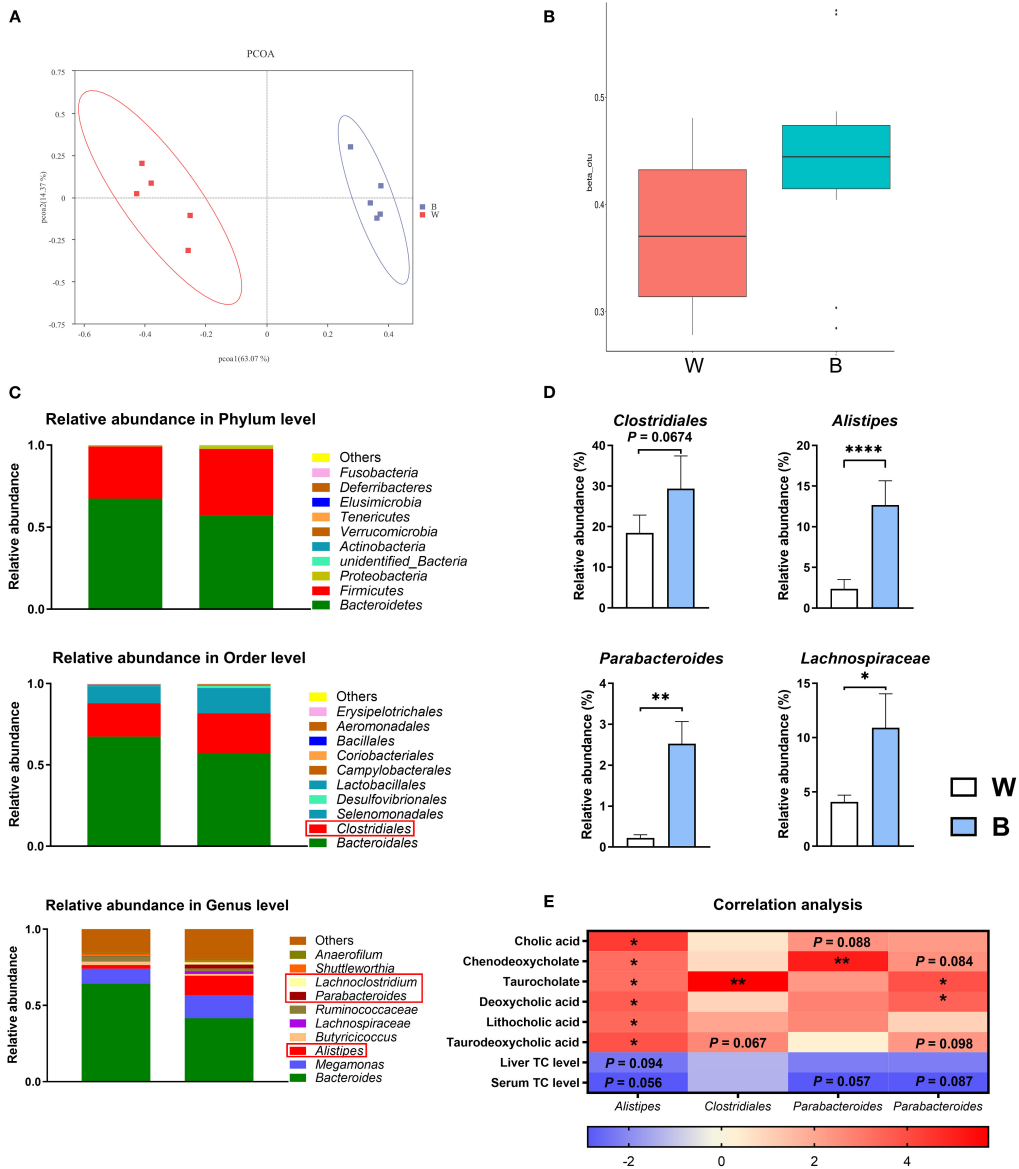
Exposure to blue light alters the intestinal microbiota. **(A)** Principal component analysis of microbial communities in the cecum from the white-light and blue-light groups (*n* = 6). **(B)** Beta diversity of microbial communities from the white-light and blue-light groups (*n* = 6). **(C)** Relative abundance of bacteria at the phylum level. OTUs with an occurrence lower than 1% are not shown. Relative abundance of the top 10 orders in each group (*n* = 6); Relative abundance of the top 10 genus in each group (*n* = 6). **(D)** Effect of blue light on the relative abundance of *Alistpes, Clostridiales, Parabacteroides*, and *Lachnospiraceae* (*n* = 6, mean with SEM). **(E)** Correlation analysis between microbiota and metabolites. Each row represents a different microorganism, and each column represents a different metabolite. Red indicates a positive correlation; blue indicates a negative correlation. Data in d were analyzed using the unpaired *t*-test, *P < 0.05, **P < 0.01, ****P < 0.0001. Data in **(E)** were analyzed using the computer non-parametric Spearman correlation, *P < 0.05, **P < 0.01.

According to a PCA analysis, the cecal microbiota was significantly different between the two groups (Figure 6A). The β diversity of the microbiota in the blue-light group was significantly higher than that in the white-light group (Figure 6B). Next, we performed an LDA effect size (LEfSe) analysis to identify changes in the relative abundance of the intestinal microbiota at the phylum, order, and genus levels (Figure 6C; Supplementary Figure 3). Notably, exposure to blue light significantly up-regulated the relative abundance of *Alistipes* (*P* < 0.0001), *Parabacteroides Clostridiales* (*P* < 0.01), and *Lachnospiraceae* (*P* < 0.05) in the cecum, and the relative abundance of *Clostridiales* also increased (*P* = 0.067; Figure 6D). Interestingly, the changes in the above bacteria are all related to the up-regulation of bile acid conversion and metabolism, and have all been found to have alleviating effects on obesity (27). Subsequently, we performed a correlation analysis on the relative abundance of intestinal microbiota with the bile acid content and cholesterol levels. The relative abundance of these bacteria is positively correlated with the content of bile acids, and negatively correlated with the level of cholesterol (Figure 6E).

These results indicate that blue light effectively shapes the intestinal microbiota of ducks and regulates the synthesis of bile acids through this process, ultimately reducing the accumulation of cholesterol.

## Discussion

Blue light refers to monochromatic light in a wavelength between 400 and 500 nm (5). Typically, short-wavelength blue light (400–450 nm) is considered to be toxic, while blue light at 450–480 nm is beneficial (13). Thus, in this study, we selected blue light with a wavelength of 460 nm to irradiate ducklings to explore its influence on the growth and development of animals compared with composite-wavelength light (380–780 nm). Our study is the first to offer experimental evidence that to blue light reduces cholesterol accumulation by altering taurine metabolism, shaping the intestinal microbiota, and promoting bile acid synthesis. Importantly, our findings demonstrate that exposure to blue light may have the potential to prevent obesity and alleviate inflammation, and may provide a new method for studying the influence of artificial light of different wavelengths as well as a foundation for research on the effects of monochromatic light in other scenarios.

Irradiation with blue light significantly reduced the weight of ducklings, but increased the weights of the breast and leg muscles, as well as their ratio. Blue light has been reported to promote broiler growth by stimulating testosterone secretion and muscle fiber growth (28). Other studies have reported that both blue light alone and a combination of blue and green light improve growth performance, bone mineral density, and fatty acid composition in ducks (17). A study on Pekin ducks revealed that blue light significantly reduced body weight and increased anxiety levels (19). These inconclusive results suggest that the effect of blue light on growth performance of animals may depend on their age, illumination intensity, light wavelength and other conditions. Further studies will be needed to clarify these points.

In this work, we found that exposure to blue light significantly reduced the IGF-1 level in serum. The elevation of IGF-1 is usually considered to be a sign of obesity and insulin resistance (29). Similarly, green light also regulates the muscle satellite cell proliferation mediated by the IGF-1 pathway (30). On the other hand, exposure to blue light significantly reduces the serum leptin and L/A levels, which suggests the optimization of lipid generation and a reduced risk of obesity (20). Significantly, the serum melatonin level is increased under exposure to blue light, possibly because blue light directly regulates hormone production through receptors in the retina and acts on the hypothalamus. The contribution of melatonin to lipid mechanism has been reported in experiments in high-fat diet mice and melatonin could up-regulate the relative abundance of *Alistipes* in the gut and thereby alleviate obesity in mice (31).

To accurately explain the role of blue light in promoting lipid metabolism and weight loss, we performed a serum metabolomics analysis in the white- and blue-light groups. Our results showed that exposure to blue light could significantly upregulate the metabolism of methionine and cysteine, thus increasing the levels of two end products: glutathione and taurine (32). Broadly, glutathione and taurine are closely related to antioxidant and lipid metabolism processes. As a powerful antioxidant, glutathione directly removes hydrogen peroxide from the organism, thereby alleviate the obesity process by decreasing lipid peroxidation (33). Therefore, glutathione depletion is usually considered to be an early warning sign of lipid peroxidation (34, 35). Simultaneously, as an isogeny product of glutathione, taurine is closely related to its synthesis. Moreover, taurine could promote SOD1 enzyme activity and inhibit mitochondrial complexes I and III to reduce ROS production (36, 37). Consequently, the increase in the antioxidant capacity of the liver and serum under irradiation by blue light is probably due to the increase in taurine and glutathione levels. In other words, the blue light-mediated up-regulation of methionine and cysteine metabolism may further promote antioxidant capacity in the duck.

The increase in the L-carnitine level is the result of the up-regulation of lysine and methionine metabolism (38). L-Carnitine is synthesized from the substrate 6-N-trimethyl-lysine; lysine residues in some proteins undergo N-methylation using S-adenosylmethionine as a methyl donor, forming 6-N-trimethyl-lysine residues (39). L-Carnitine is closely related to muscle production and fat metabolism. It is also well-known as a fat-burning agent, but it is rarely used in animal husbandry (40). It has been reported in the literature that the addition of L-carnitine to the summer diet improves the growth performance of ducks (41). Notably, L-carnitine significantly improved nutrient digestibility coefficients in ducks. Under blue light the serum glutamine level also tends to be up-regulated. Recent studies have reported that glutamine activated an mTOR signaling pathway and stimulated the proliferation of muscle satellite cells (42). Meanwhile, glutamine could also improve the oxidative damage in broiler thin muscle (43, 44). A correlation analysis of these two metabolites in serum and muscle development showed that blue light increased the contents of L-carnitine and L-glutamine by up-regulating the metabolism of methionine and cysteine, thereby promoting muscle development in ducks.

This study also demonstrated that blue light had an inhibitory effect on inflammatory cytokines, which is mostly reflected in the promotion of wound healing *in vitro*. Indeed, Lehrl et al. (12) reported that exposure to blue light attenuated the cognitive impairment caused by neuroinflammation. In this study, blue light also promoted anti-inflammatory capacity inside the body. We speculate that blue light may inhibit inflammatory cytokines through multiple pathways: i.e., *via* hormonal regulation (e.g., leptin, adiponectin, and melatonin) or the up-regulation of antioxidant levels (45–47).

In addition to the aforementioned antioxidant and cytoprotective effects, recent studies have shown that the use of taurine supplements can effectively reduce obesity (48). With the promotion of autophagy in adipocytes (49), taurine can inhibit the production of white adipose tissue (50, 51). More importantly, Guan and Miao (52) reported that taurine supplementation reduced total cholesterol and triglyceride levels in serum, thereby lowering blood pressure and improving blood lipids. Our research found that exposure to blue light can also significantly reduce serum and liver cholesterol levels, which may be related to changes in the taurine content. This result also suggests that blue-light regulation of cholesterol metabolism may play a pivotal role in achieving body weight loss.

After cholesterol is metabolized, bile acids are produced in the liver. As a downstream metabolite of cholesterol, bile acid can be classified as primary bile acid or secondary bile acid (53). The production, metabolism, and circulation of bile acids is a complex process. In the liver, cholesterol is converted into primary bile acids before it passes into the intestine. In the intestine, bacteria that produce bile acid hydrolase (BSH) convert primary bile acids into secondary bile acids. The interaction between intestinal flora and bile acids is homeostatic and synergetic. The intestinal microbiota affects the production and enterohepatic circulation of bile acids, while bile acids could also affect the gut microbe composition (54). Bile acid plays a key role in nutrient absorption and distribution, metabolism regulation, and homeostasis (55). Bile acid chelator supplement has even been used for the treatment of obesity (56).

Therefore, after noting the down-regulated cholesterol levels following exposure to blue light, we subsequently measured bile acid levels in serum. Interestingly, both primary and secondary bile acid levels tended to be up-regulated. The higher content of primary bile acids is predictable because taurine plays an indispensable role in converting cholesterol to bile acids. Taurine activates the expression of the *CYP7A*1 gene to promote cholesterol conversion and could be used as a precursor binding to cholesterol to form bile salts (57, 58). In addition to promoting lipid digestion, bile acids are also specific receptors for farnesol X (FXR). After FXR is activated, it can reduce the accumulation of cholesterol and triglycerides and relieve obesity (59–61). Our results indicated that exposure to blue light might control body weight by regulating cholesterol and bile acid homeostasis.

Despite these findings, secondary bile acid levels also tended to increase after blue-light irradiation. Generally, gut microbes play an integral role in converting primary bile acids to secondary bile acids (62). The results of 16S rRNA sequencing also showed that exposure to blue light significantly increased the abundance of *Clostridiales, Alistipes, Parabacteroides*, and *Lachnospiraceae* in the duck cecal microbiota. *Clostridium scindens* and *Extibacter muris* have been reported to metabolize primary bile acids to deoxycholic acid in mice (63). Moreover, as BSH-produced bacteria, *Clostridiales* play an essential role in converting and refluxing bile acids (64). In this experiment, exposure to blue light significantly increased the relative abundance of *Clostridiales*, and the content of deoxycholic acid was increased 1.92-fold. The relative abundance of *Alistipes* was also strongly up-regulated and that in the blue-light group reached 5.4-fold that in the white-light group, which may be due to the unique resistance of *Alistipes* to bile acids (65). The up-regulation of *Alistipes* may also be related to the alleviation of metabolic disorders and the reduction of cholesterol content. Various studies have explored the efficacy of *Alistipes* against obesity. For instance, *Alistipes* could reduce lipid metabolism disorder induced by a high-fat diet in mice by producing acetic acid (31). The up-regulation of probiotics such as *Alistipes* also stimulates CYP7A and promotes cholesterol conversion to bile acids (66). In addition, studies have shown that a high abundance of *Alistipes* can increase the intestinal absorption of cholesterol and reduce the synthesis of cholesterol in the liver (67). Thus, *Alistipes* seems to be able to directly use cholesterol to promote its own proliferation and further reduce the accumulation of cholesterol in the liver and serum. Furthermore, *Parabacteroides* has been reported to improve succinate and secondary bile acid levels and play critical roles in modulating host metabolism (68). In our study, the relative abundance of *Parabacteroides* in the blue-light group also increased significantly. In addition, *Lachnospiraceae*, as a kind of butyrate-producing probiotic, promotes the growth and development of the intestines (69). Irradiation with blue light increases the relative length of the intestine, which is probably the result of the increase in the relative abundance of short-chain fatty acid-producing bacteria, and the increase in the β diversity of the gut microbes in the blue-light group also increases the stability of the gut microecology (16, 70, 71).

More importantly, previous studies have found that the relative abundance of *Lachnospiraceae* in the intestines of obese mice is significantly down-regulated (72). These results suggested that blue light could shape the intestinal microbiota and regulate the changes in the relative abundance of BSH-related bacteria (e.g., *Clostridiales, Alistipes, Parabacteroides*, and *Lachnospiraceae*). The alteration of microbiota and metabolites significantly contributes to the variation of bile acid and lipid metabolism, resulting in a reduction in cholesterol accumulation and the loss of weight in ducks. However, more evidence is needed.

We concluded that blue light could regulate cysteine and methionine metabolism and shape the intestinal microbiota based on our experimental results. Our findings also opened up a new direction of conjecture (regarding the brain-gut axis) for exploring the mechanism by which blue light regulates lipid metabolism.

## Conclusion

The present study applied non-targeted metabolomics and an intestinal microbiota diversity analysis to investigate the effects of exposure to blue light on duck metabolism. Blue light triggered cysteine and methionine metabolism, and improved lipid metabolism. By increasing bile acid production and bile acid hydrolase-producing bacteria, exposure to blue light reduced the accumulation of cholesterol in the host. These results highlighted the change in metabolic function and the intestinal microbiota in blue light exposure-induced weight loss. This study also provides a new experimental approach for the application of blue light in animal husbandry.

## Data Availability Statement

The datasets presented in this study can be found in online repositories. The names of the repository/repositories and accession number(s) can be found below: NCBI; PRJNA744139.

## Ethics Statement

The animal study was reviewed and approved by Animal Care and Use of South China Agricultural University (No. 20110107-1, Guangzhou, China).

## Author Contributions

WW, LY, and JC designed the study. JC, YL, YF, and DX acquired the data and performed experiments. YZhu, HY, YF, and JQ helped conduct animal experiments. DX, XJ, GM, SW, and YZhao advised on data analysis. WW and DX wrote the manuscript. All authors read and approved the manuscript.

## Funding

This study was sponsored by the National Nature Science Foundation of China (32072751), the National Key Research Program (2016YFD0500509-07), Natural Science Foundation of Guangdong Province (2019B1515210012), the China Agriculture Research System (CARS-42-15), and Modern Agricultural Industrial Technology System Innovation Team of Guangdong Province (2020KJ137).

## Conflict of Interest

XJ is employed by Moon (Guangzhou) Biotech Co. Ltd. SW and YF are employed by Guangdong Haida Group Co. Ltd. YZhao is employed by Gold Coin Feedmill (Dong Guan) Co. Ltd. JQ is employed by Cofco Feed (Foshan) Co. Ltd. The remaining authors declare that the research was conducted in the absence of any commercial or financial relationships that could be construed as a potential conflict of interest.

## Publisher's Note

All claims expressed in this article are solely those of the authors and do not necessarily represent those of their affiliated organizations, or those of the publisher, the editors and the reviewers. Any product that may be evaluated in this article, or claim that may be made by its manufacturer, is not guaranteed or endorsed by the publisher.
