# Supplementary material for: Combined Analysis of the Effects of Exposure to Blue Light in Ducks Reveals a Reduction in Cholesterol Accumulation Through Changes in Methionine Metabolism and the Intestinal Microbiota
Source: Front Nutr. 2021 Nov 25;8:737059. doi: 10.3389/fnut.2021.737059 (PMC8656972; doi:10.3389/fnut.2021.737059)
Supplement: Supplementary file 1 [file Image_1.pdf]

Supplementary Material

Supplementary Figures

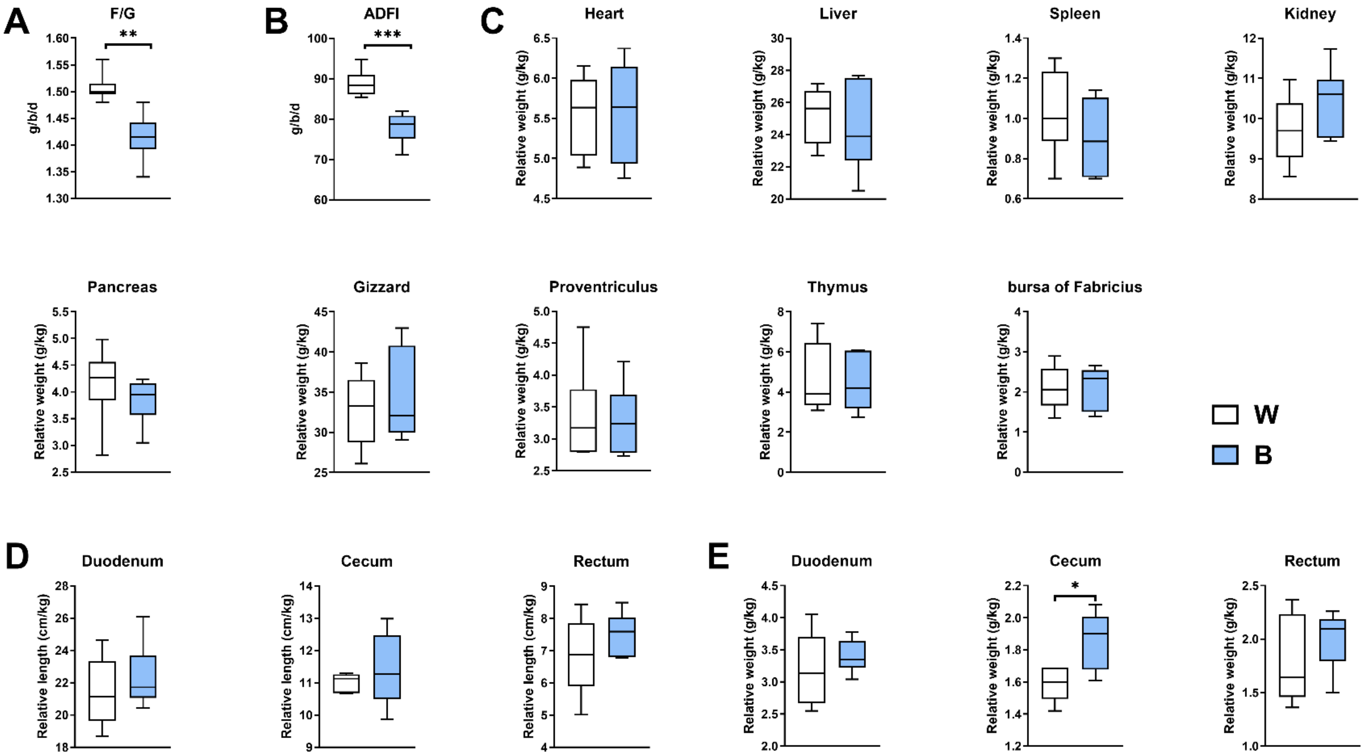

Supplementary Figure 1. (A - B) Effect of 460 nm LED blue light exposure treatment on growth performance in ducks. (C) Effect of 460 nm LED blue light exposure treatment on organ index in ducks. (D - E) Effect of 460 nm LED blue light exposure treatment on the relative length and weight of the intestine.

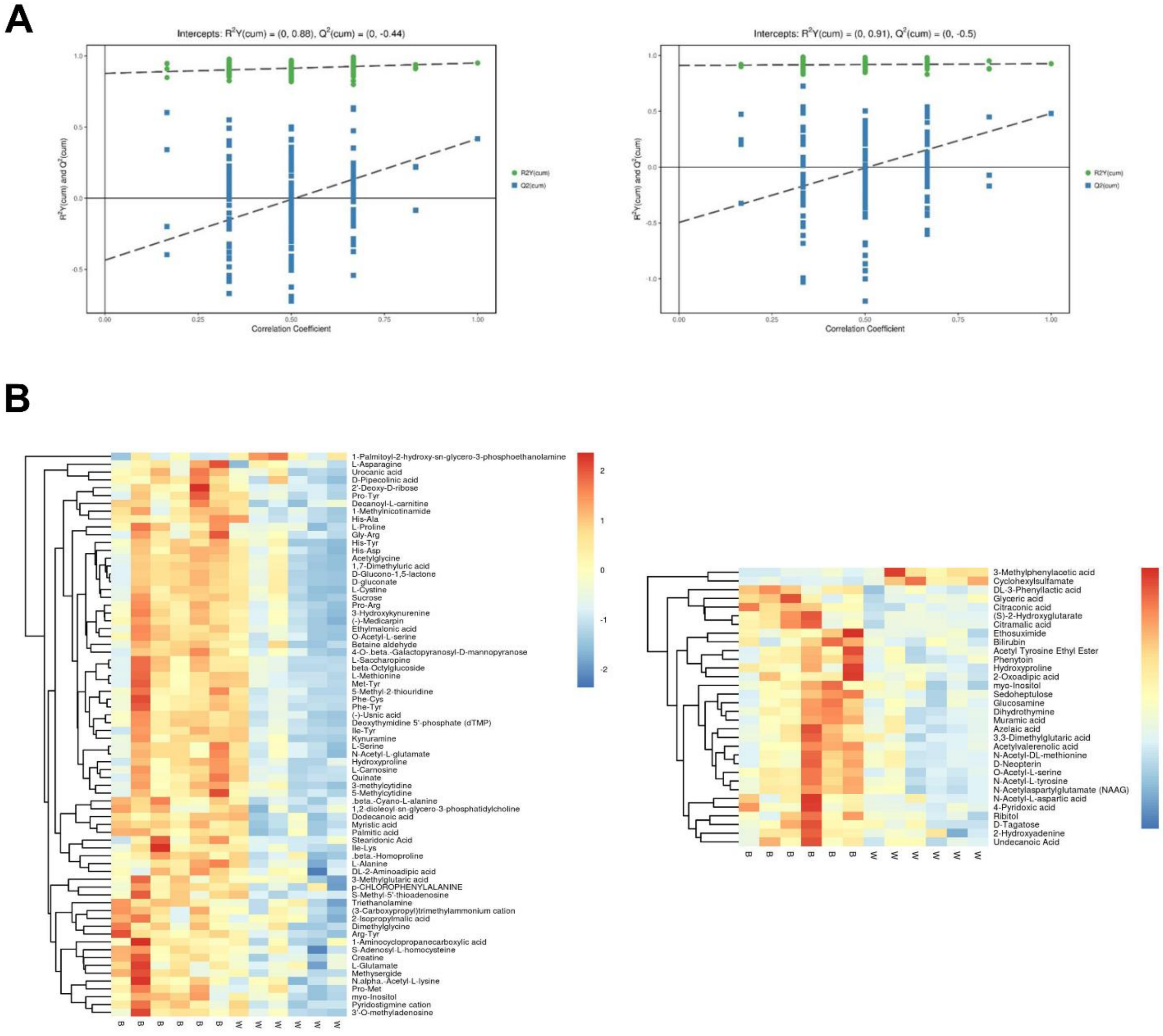

Supplementary Figure 2. (A) Permutation test of OPLS-DA model showing the stability of the model (n = 6). (B) Heatmaps represent the differential metabolites identified between the blue light exposure group vs the white light exposure group (n = 6).

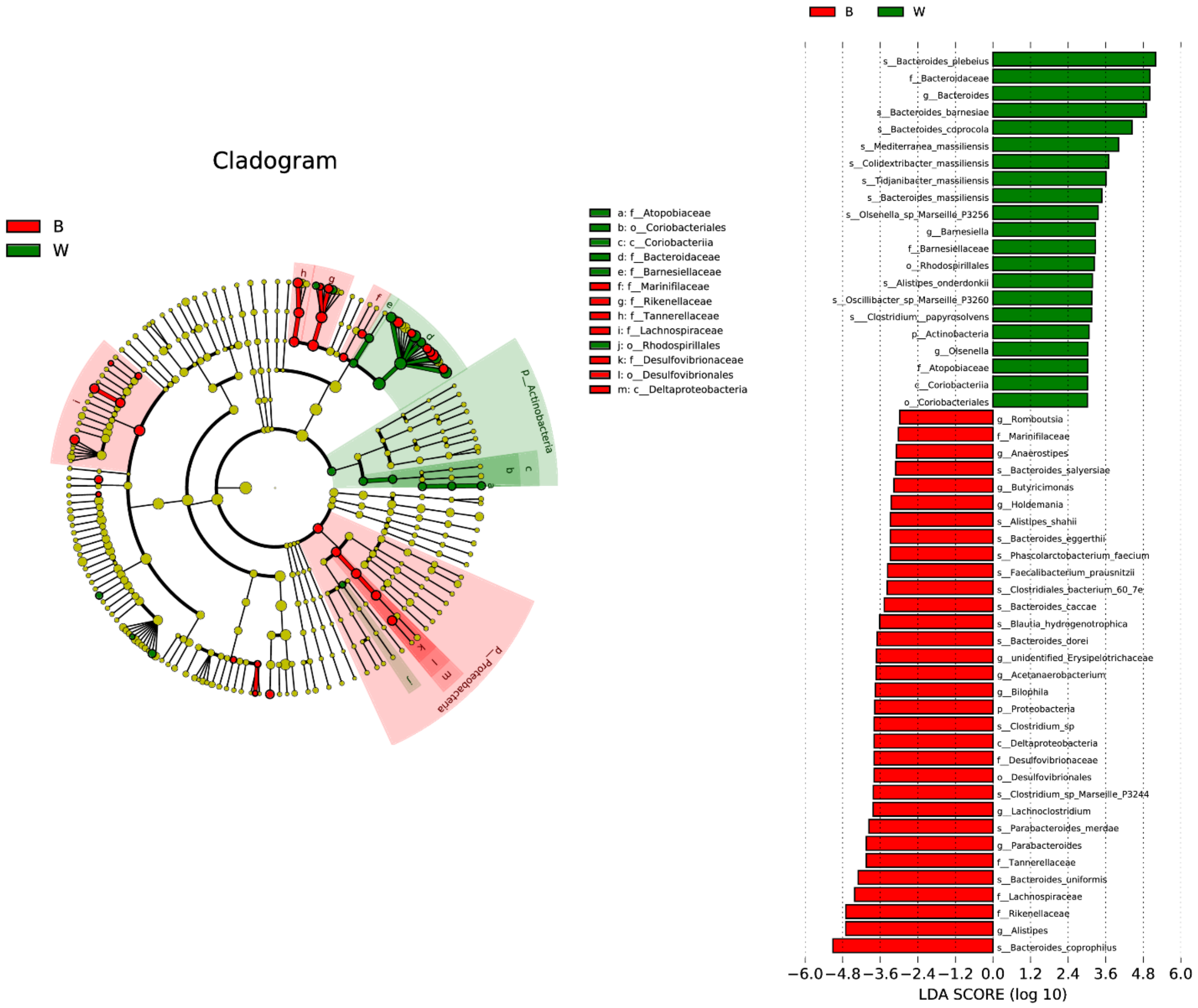

Supplementary Figure 3. LDA Effect Size analysis of microbial communities in the cecum from white light exposure groups and blue light exposure (n=6).
